# Micro spies from the brain to the periphery: new clues from studies on microRNAs in neuropsychiatric disorders

**DOI:** 10.3389/fncel.2014.00075

**Published:** 2014-03-11

**Authors:** Elisabetta Maffioletti, Daniela Tardito, Massimo Gennarelli, Luisella Bocchio-Chiavetto

**Affiliations:** ^1^Genetic Unit, IRCCS Centro S. Giovanni di Dio FatebenefratelliBrescia, Italy; ^2^Department of Molecular and Translational Medicine, University of BresciaBrescia, Italy; ^3^Dipartimento di Scienze Farmacologiche e Biomolecolari, Università degli Studi di MilanoMilano, Italy; ^4^Neuropsychopharmacology Unit, IRCCS Centro S. Giovanni di Dio FatebenefratelliBrescia, Italy

**Keywords:** microRNA, schizophrenia, major depression, bipolar disorder, Alzheimer disease, Parkinson disease, genetic variation, SNP

## Abstract

microRNAs (miRNAs) are small non-coding RNAs (20–22 nucleotides) playing a major role in post-transcriptional regulation of gene expression. miRNAs are predicted to regulate more than 50% of all the protein-coding genes. Increasing evidence indicates that they may play key roles in the biological pathways that regulate neurogenesis and synaptic plasticity, as well as in neurotransmitter homeostasis in the adult brain. In this article we review recent studies suggesting that miRNAs may be involved in the pathophysiology of neuropsychiatric disorders and in the action of psychotropic drugs, in particular by analyzing the contribution of genomic studies in patients' peripheral tissues. Alterations in miRNA expression have been observed in schizophrenia, bipolar disorder, major depression, Parkinson's disease, Alzheimer's disease and other neuropsychiatric conditions. In particular, intriguing findings concern the identification of disease-associated miRNA signatures in peripheral tissues, or modifications in miRNA profiles induced by drug treatments. Furthermore, genetic variations in miRNA sequences and miRNA-related genes have been described in neuropsychiatric diseases. Overall, though still at a preliminary stage, several lines of evidence indicate an involvement of miRNAs in both the pathophysiology and pharmacotherapy of neuropsychiatric disorders. In this regard, the data obtained in peripheral tissues may provide further insights into the etiopathogenesis of several brain diseases and contribute to identify new biomarkers for diagnostic assessment improvement and treatment personalization.

## Introduction

**Figure 1:**
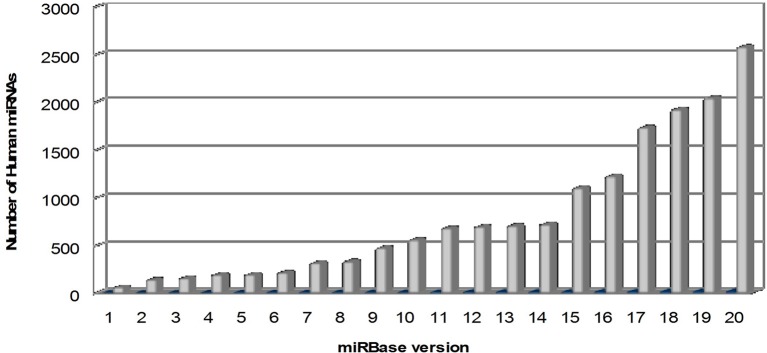
**Overtime trend in the number of human microRNAs annotated in miRBase**. The number of mature human microRNAs annotated in miRBase database (Kozomara and Griffiths-Jones, 2014; http://www.mirbase.org) is continuously growing, starting from few dozens in the first release (2002) to more than 2500 in the last release (version 20, June 2013).

microRNAs (miRNAs) are a large family of conserved small (20–22 nucleotides) non-coding RNAs, with a key role in the post-transcriptional regulation of gene expression. In mammals, miRNAs are predicted to control the activity of ~50% of all the protein-coding genes. Their discovery dates back to 1993 with the identification of lin-4, a small ribonucleotide molecule involved in the regulation of “larva to adult switch” in *C. elegans* (Lee et al., 1993). miRBase, the primary online repository for all miRNA sequences, continuously upgrades the data on newly identified miRNAs and nowadays, at its 20th release (June 2013), it annotates 2578 human mature miRNAs and 1872 precursors (Kozomara and Griffiths-Jones, 2014; http://www.mirbase.org) (Figure 1).

**Figure 2:**
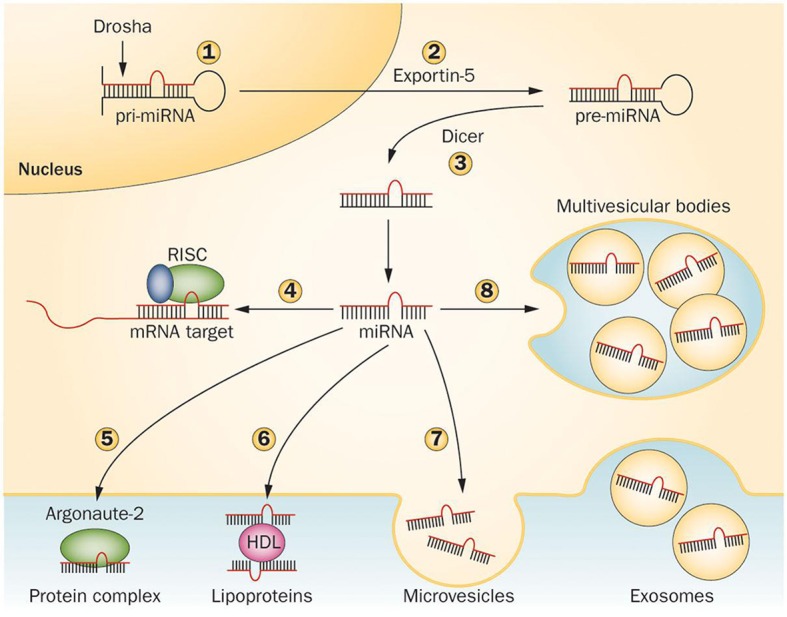
**Mechanisms regulating microRNA processing and release**. Pri-miRNAs are cleaved in the nucleus by Drosha (1) to generate pre-miRNAs, then exported in the cytoplasm by Exportin-5 (2) and further cleaved by Dicer to produce 21–23 nucleotide duplexes (3). One strand of the miRNA duplex can either associate to the RISC complex and guide translational repression of target mRNAs (4) or be released by the cells. In the latter case, the mature miRNA binds to RNA-binding proteins, such as Argonaute-2 (5) or to lipoproteins (6). Alternatively, miRNAs can be loaded in microvesicles formed by plasma membrane blebbing (7) or in exosomes that are released in the extracellular space upon exocytic fusion of multivesicular bodies with the plasma membrane (8). Abbreviations: miRNA, microRNA; pre-miRNA, miRNA precursor; pri-miRNA, primary miRNA transcript; RISC, RNA-induced silencing complex. Figure reprinted by permission from Macmillan Publishers Ltd: Nature Reviews Endocrinology, Guay C. and Regazzi R. Circulating microRNAs as novel biomarkers for diabetes mellitus. 9, 513–521 (September 2013). doi: 10.1038/nrendo.2013.86.

miRNAs are transcribed in the nucleus by RNA polymerase II to primary miRNA (pri-miRNA) transcripts, double-stranded stem loop structures of 100–1000 nucleotides in length, then processed to >60–70 nucleotide precursors (pre-miRNAs), by a complex containing the RNAse-III type endonuclease Drosha and its cofactor DGCR8, as well as other cofactors. Pre-miRNAs are then exported in the cytoplasm by exportin-5 and cleaved in a ~20 bp miRNA/miRNA^*^ duplex by the RNase-III type enzyme Dicer and its cofactor TRBP. In mammals, Dicer is supported by Argonaute 2 (Ago2), a RNaseH-like endonuclease that cleaves the 3' arms of pre-miRNAs, thus generating mature miRNAs. The “right” strand of the miRNA duplex is then loaded into the RNA-induced silencing complex (RISC), whereas the other strand (miRNA^*^) is released and degraded, although in some cases both strands can associate with RISC to target distinct sets of mRNAs (Schwarz et al., 2003; Davis and Hata, 2009; Breving and Esquela-Kerscher, 2010; Krol et al., 2010; O'Carroll and Schaefer, 2012) (Figure 2).

miRNAs regulate protein synthesis post-transcriptionally by base-pairing to target mRNAs. Generally, miRNAs inhibit protein synthesis either by repressing translation or by inducing deadenylation and degradation of target mRNAs, but were also reported to activate translation (Chekulaeva and Filipowicz, 2009; Huntzinger and Izaurralde, 2011). Individual miRNAs have the potential to target hundreds of different mRNAs, and a single mRNA can be modulated by several different miRNAs, thus implying a coordinate and fine-tuned regulation of protein expression in a cell and even in particular cell compartments (Krol et al., 2010; O'Carroll and Schaefer, 2012).

Many miRNAs are expressed in a tissue-specific or developmental stage-specific manner, thereby contributing to cell type-specific profiles of protein expression. Functional studies indicate that miRNAs participate in the regulation of almost every cellular process and therefore it is not surprising that changes in their expression or function are associated with many human pathologies (Sayed and Abdellatif, 2011; Chan and Kocerha, 2012; Pasquinelli, 2012), as cancer (Farazi et al., 2013; Profumo and Gandellini, 2013) and cardiovascular diseases (Madrigal-Matute et al., 2013; Papoutsidakis et al., 2013).

In the past few years growing evidence has supported a key role for miRNAs in central nervous system (CNS) development and homeostasis. It has been reported that almost 50% of all the identified miRNAs are expressed in the human brain, with putative target genes regulating synaptogenesis and other basic neuronal processes (Ziats and Rennert, 2013). A role for miRNAs in neurogenesis, neuronal differentiation and survival, as well as in neuroplasticity, is now well established, although further work is needed to better clarify these aspects (Smalheiser and Lugli, 2009; Siegel et al., 2011; Olde Loohuis et al., 2012).

The unique mode of functioning of miRNAs, that is, the ability of a single miRNA to target several different mRNAs often belonging to specific functional networks, has prompted research toward the study of the potential involvement of miRNAs in the pathogenesis and pharmacotherapy of neurologic and psychiatric disorders (Kolshus et al., 2013; Tardito et al., 2013).

First evidence in post-mortem brain studies showed an overall decrease of miRNA expression in the prefrontal cortex (PFC) of schizophrenic (SCZ) subjects (Perkins et al., 2005, 2007; Miller et al., 2012). Other authors described an increase in miRNA expression in temporal regions of SCZ patients, associated to a dysregulation of the biogenesis cofactor DGCR8, *inter alia* mapped in the 22q11 Di George syndrome critical region, one of the candidate susceptibility loci for SCZ (Beveridge et al., 2008, 2010). Elevated miRNA expression and DICER1 mRNA increase were observed also by Santarelli et al. (2011) in dorsolateral PFC of SCZ post-mortem brains. Furthermore, alterations in miRNA levels were evidenced in post-mortem PFC from bipolar patients (BD) (Kim et al., 2010; Moreau et al., 2011). Notably, most of the differentially expressed miRNAs were downregulated in both the SCZ and BD groups relative to controls, in line with previous results (Perkins et al., 2007), but only a few of them were in common among the various studies. More recently, Banigan et al. (2013) reported an increase in exosomal miRNA content in SCZ post-mortem brains. Finally, an overall decrease in miRNA expression was observed in PFC of depressed suicide committers, with significant modifications of 21 miRNAs (Smalheiser et al., 2012).

Regarding a possible involvement of miRNAs in the action of psychotropic drugs, Zhou et al. (2009) showed that chronic treatment with mood stabilizers induced significant modifications of miRNA expression in the rat hippocampus. The effects of lithium on miRNA expression were confirmed also by a study in lymphoblastoid cell lines (LCLs) from BD patients (Chen et al., 2009). First preclinical studies on antidepressant drug effects suggested a role for miR-16 in the mechanism of action of fluoxetine; specifically, miR-16 appeared to create new serotonin sources in the brain through the switch of noradrenergic neurons toward a serotonergic phenotype (Baudry et al., 2010). Treatments with fluoxetine and desipramine, two antidepressants with a different primary mechanism of action, were reported to induce early and time-associated miRNA modulation in rat hippocampus (Pelizzari et al., 2012). Acute treatment with ketamine (an NMDA receptor antagonist shown to induce a rapid and sustained antidepressant effect), electroconvulsive shock therapy and chronic fluoxetine treatment were described to reverse the changes in rat hippocampal miRNA expression induced by early life stress (O'Connor et al., 2013). In a genome-wide miRNA investigation conducted on LCLs screened for growth inhibition by paroxetine, Oved et al. (2012) observed a differential expression of 6 miRNAs in paroxetine-sensitive cells, suggesting that these miRNAs could represent tentative SSRI response biomarkers. Finally, a modulation of small subsets of miRNAs regulating metabolic pathways was also reported after treatment with different antipsychotics, supporting possible associations with drug side effects (Santarelli et al., 2013).

Accumulating evidence indicates also in Alzheimer's disease (AD) brains a dysregulation of specific miRNAs, several of which potentially involved in the regulation of key disease genes (see for review: Junn and Mouradian, 2012; Tan et al., 2013). Among them, particularly interesting is the miR-29 cluster, which was significantly downregulated in AD patients in whom BACE-1 (β-amyloid cleavage enzyme 1) protein was aberrantly increased (Hébert et al., 2008; Nunez-Iglesias et al., 2010). Moreover, a decrease of miR-107 in AD brains was reported, paralleled by an increase of BACE-1 mRNA levels (Wang et al., 2008a, 2011; Nelson and Wang, 2010). Another dysregulated miRNA in AD brains is miR-132, which was described to be differentially expressed also in frontotemporal lobar degeneration (FTLD), together with 2 other miRNAs belonging to the same cluster (miR-132^*^ and miR-212) (Chen-Plotkin et al., 2012; Hébert et al., 2013). Among the top target mRNAs of both miR-132 and miR-212 there is TMEM106B, a gene linked to FTLD by a genome-wide association study (GWAS) (Van Deerlin et al., 2010). Concerning Parkinson's disease (PD), decreased brain expression levels of miR-34b and miR-34c were observed (Miñones-Moyano et al., 2011), potentially affecting key pathways in PD pathogenesis, such as mitochondrial dysfunction, and reducing DJ1 and Parkin levels. Moreover, miR-133 was identified as deficient in PD midbrain tissue showing neuronal loss (Kim et al., 2007). Also *in vitro* studies supported the involvement of miRNAs in PD; as an example, miR-7 and miR-153 were shown to downregulate the expression of α-synuclein, one of the key genes implicated in PD etiopathogenesis (Junn et al., 2009; Doxakis, 2010).

### microRNAs as biomarkers in peripheral tissues

Besides their presence in cells, miRNAs were also observed in a highly stable, cell-free form (Cortez et al., 2011). Indeed, a number of studies have detected miRNAs in several peripheral biological matrices, including whole blood, plasma, serum, cerebrospinal fluid (CSF), saliva, and others (Cogswell et al., 2008; Mitchell et al., 2008; Park et al., 2009; Hanke et al., 2010; Zubakov et al., 2010).

Although it is clear that miRNAs function as a mechanism for post-transcriptional regulation, it has not been conclusively proven whether their presence in body fluids is simply a by-product of cell degradation or whether are they actively secreted into the body fluids to mediate intercellular gene regulation. A body of evidence supports the hypothesis that miRNAs can be actively and selectively secreted; for example, miR-1246 and miR-451 were found to be released by the breast cancer cell line MCF-7, but not by the non-malignant mammary epithelial breast cell line (Pigati et al., 2010). Also in support of active secretion is the appropriate packaging of miRNAs to facilitate circulation and to protect them from degradation in body fluids. miRNAs in serum are resistant to circulating ribonucleases and severe physico-chemical conditions, such as extended storage, freeze-thawing and extreme pH (Chen et al., 2008; Mitchell et al., 2008). As described in Figure 2, there are three known ways by which miRNAs are packaged: in lipid microvesicles, such as exosomes and apoptotic bodies; bound by RNA-binding proteins, such as nucleophosmin 1 and Argonaute 2; and associated with high-density lipoproteins (Wang et al., 2010a; Arroyo et al., 2011). Similarly to hormones and cytokines, secreted miRNAs might serve as signaling molecules of cell-to-cell communication (Valadi et al., 2007), and their packaging also facilitates their transfer between individuals, as exemplified by the case of immune-related miRNAs in breast milk in the first 6 months of lactation, showing that packaged miRNAs could be absorbed orally and not digested (Iguchi et al., 2010).

Recently, studies suggested that miRNAs in plasma and serum might derive from circulating blood cells under healthy conditions, but could be released from pathological tissues during an illness (Chen et al., 2008; Fichtlscherer et al., 2010). The correlation between circulating miRNAs and tissue miRNAs suggests that miRNAs in human fluids might serve as biomarkers for various diseases (Skog et al., 2008; Laterza et al., 2009; Zeng et al., 2011). Some of the innate properties of miRNAs make them highly attractive as potential biomarkers: miRNAs can be readily detected in small volume samples using specific and sensitive quantitative real-time PCR (qRT-PCR), and their levels in plasma and serum are stable. Moreover, blood collection is a common and easy clinical procedure, and different individuals within the same species display similar levels of circulating miRNAs. However, a prerequisite to use circulating miRNAs as diagnostic and prognostic biomarkers is the ability to quantify them in different matrices (plasma, serum, CSF, whole blood) with an adequate sensitivity and precision. The quality of miRNA measurements with different techniques might be associated to many variables, including those related to preanalytic variants, such as specimen collection, RNA extraction efficiency and technical issues related to data analysis and normalization (Kroh et al., 2010). For example, there is a risk of cellular contamination during CSF collection with lumbar puncture and plasma and serum preparation; moreover, the anticoagulant used might influence the results of the analyses, since heparin impedes a qRT-PCR step (Boeckel et al., 2013). Furthermore, there may be an individual variability in both the protein and lipid content in serum and plasma specimens that could affect the efficiency of RNA extraction. Finally, there is no consensus on suitable small RNA reference genes that could be used as internal controls for normalization in different biological fluids (Mitchell et al., 2008; Kroh et al., 2010).

### Genetic variants in microRNA-related genes

Two major classes of genetic variants in miRNA-related genes have been documented: single nucleotide polymorphisms (SNPs) and copy number variations (CNVs). SNPs are small genetic variations in chromosomal DNA sequences in which a single nucleotide is substituted by one of the other three nucleotides. SNPs are the most common form of variation present in the human genome (~10–30 million SNPs with a frequency >1% in the human population, occurring on average every 100–300 bases). The availability of high-throughput technologies investigating the genome led to the demonstration that a large number of genomic sequences, many of which encompass entire genes, vary in copy number among individuals. These deletions and duplications, referred to as CNVs, are more common in the general population than ever imagined before. Beside population-specific, common CNVs, there are rare, disease-causing CNVs, which constitute an important class of genetic variability in mendelian and multifactorial disorders. Both SNPs and CNVs in miRNA-related genes are underrepresented compared with the reference human genome, suggesting possible negative selection (Duan et al., 2009; Felekkis et al., 2011; Marcinkowska et al., 2011). In contrast, the number of miRNA target genes in polymorphic CNVs is higher than in non-CNV regions, suggesting that genes integral to polymorphic CNVs are more likely to be regulated by miRNAs, in order to counteract their expression changes due to copy number variability of the region in which they reside (Felekkis et al., 2011). Multiple cancer studies show that miRNAs integral to CNVs demonstrate gain or loss at the genomic level, and are associated with expression changes for ~10–20% of miRNAs (Schiffman et al., 2011; Shim et al., 2012).

Genetic variants in miRNA-related genes include variations in miRNA/pri-/pre-miRNA sequences, in miRNA biogenesis and machinery genes, as well as in the 3'-UTR of target genes, where mature miRNAs are bound. Therefore, these variants can affect the transcription of pri-miRNAs, the processing and maturation of pre-miRNAs and miRNA-mRNA interaction.

The initial demonstration that miRNA-related genetic variants can affect disease phenotype was given by Abelson et al. (2005), who found that a mutation in miR-189 binding site of SLITRK1 gene was associated with Tourette's syndrome. Since then, several studies have identified associations between polymorphisms, mainly SNPs, influencing miRNA function and different human disorders, going from PD to multiple forms of cancer (Sethupathy and Collins, 2008).

The identification of miRNA SNPs has greatly improved in the last few years. The first efforts aimed at the selection of these variants were conducted by Muiños-Gimeno et al. (2010) and by Duan et al. (2009), who respectively identified 24 and 187 SNPs in miRNA/pre-miRNA sequences, employing the by now old miRBase versions 7.1 and 13.0. More recently, thanks to the 1000 genomes project (http://www.1000genomes.org), many other variants have been identified; so far, more than 1000 miRNA SNP have been annotated (Han and Zheng, 2013). Many online databases collecting miRNA SNPs, more or less up-to-date, are available; some of them also offer information about the association with various diseases [see for example MicroSNiPer (http://epicenter.ie-freiburg.mpg.de/services/microsniper/), Patrocles (http://www.patrocles.org/), Polymirts (http://compbio.uthsc.edu/miRSNP/) and mirSNP (http://202.38.126.151/hmdd/mirsnp/search/)].

In this narrative review we present a wide overview of recent studies analyzing both disease alterations in miRNA expression levels in patients' peripheral matrices and associations with miRNA-related genetic variants.

## Expression studies in human peripheral tissues

### Expression studies in psychiatric disorders

#### Schizophrenia

Gardiner et al. (2012) analyzed the global miRNA expression in peripheral blood mononuclear cells (PBMCs) from SCZ patients compared to healthy controls and identified an expression profile significantly associated with SCZ; many of the differentially-expressed miRNAs were found to be part of a large cluster on the imprinted DLK1-DIO3 region on chromosome 14q, suggesting a possible significant underlying genetic or epigenetic alteration associated with this disease. To gain an appreciation of the biological implications of the disease-associated changes, the authors also examined predicted miRNA targets, identifying many pathways related to neural functions, such as axon guidance, regulation of the actin cytoskeleton, long-term potentiation, long-term depression, neuroactive ligand–receptor interaction, focal adhesion and neurotrophins. A similar study was conducted by Lai et al. (2011) by evaluating miRNA expression profiles in white blood cells (WBCs) from SCZ subjects. A 7-miRNA signature was significantly associated with SCZ diagnosis and its clinical characteristics, such as symptoms, neurocognitive performances and neurophysiological functions, showing a high discriminating accuracy. The predicted target genes for the identified miRNAs were shown to pertain to pathways involved in nervous system development and function, such as cyclin-dependent kinase 5 (Cdk5), Notch and dopamine receptor signaling. A candidate miRNA approach was instead employed by Shi et al. (2012), who measured in SCZ patients' serum the levels of 9 miRNAs, selected on the basis of previously published studies, since they had been shown to be implicated in SCZ or predicted to target SCZ-related genes. Among them, 5 were shown to be differentially expressed. Changes in miRNA expression were also observed to be induced by and/or implicated in effective antipsychotic treatment: 2 miRNAs were downregulated after a 1-year treatment with risperidone in plasma from first-episode SCZ patients, all of which had achieved remission (Liu et al., 2013).

#### Bipolar disorder

Only one expression study was conducted on BD for one candidate miRNA (Rong et al., 2011). In plasma from drug-free manic patients, miR-134 was shown to be downregulated compared to controls; consistently, its level increased after a 4-weeks treatment with different combinations of antypsychotics and/or mood stabilizers. Both in drug-free and in medicated patients (2 and 4 weeks), miR-134 levels were negatively correlated with manic symptoms, assessed through BRMS scores.

#### Major depression and anxiety

The first study on peripheral miRNA expression in drug-free patients suffering from MD was conducted by Bocchio-Chiavetto et al. (2013), by evaluating the changes in global miRNA levels in whole blood after a 12-weeks effective treatment with the antidepressant drug escitalopram (a SSRI). A modulation was observed for 30 miRNAs; interestingly, target gene prediction and pathways analysis showed that these miRNAs might be implicated in several pathways associated with brain functions, such as neuroactive ligand–receptor interaction, axon guidance, long-term potentiation and depression, supporting the hypothesis of their involvement in the antidepressant mechanism. Belzeaux et al. (2012), by means of a global analysis, reported a differential expression of 14 miRNAs in PBMCs from non drug-free MD patients compared to controls. Putative interactions between the dysregulation in miRNAs and in mRNAs, identified through a parallel expression analysis, have been subsequently recognized. Moreover, after an effective 8-weeks treatment with different classes of antidepressant drugs, in mono- or polytherapy, a modulation was observed for 8 miRNAs. Finally, Li et al. (2013) reported in the serum of MD patients an upregulation of 2 miRNAs which had been previously described to decrease *in vitro* protein levels of brain-derived neurotrophic factor (BDNF), a neurotrophin widely implicated in MD.

Although no expression study on anxiety-related disorder in human peripheral tissues is available, it was reported that stressful conditions due to academic examination induce an enhancement in blood levels of specific miRNAs, in particular miR-16, miR-144/144^*^ and miR-26b (Katsuura et al., 2012; Honda et al., 2013).

Table 1 summarizes the above-reported expression studies in psychiatric disorders, with indication of samples, methodologies, and main results.

**Table 1:** **microRNA expression studies in psychiatric disorders**.

**Disease**	**Sample**	**Tissue**	**Method**	**Main finding**	**Reference**
Schizophrenia	112 SCZ vs. 76 CTRL	PBMCs	miRNA array Illumina (miRBase v. 9.1), qRT-PCR	↓ miR-31, miR-99b, miR-107, miR-134, miR-431, miR-433, miR-487b	Gardiner et al., 2012
	90 SCZ vs. 60 CTRL	WBCs	TaqMan Low Density Array v. 1.0	↑ miR-34a, miR-449a, miR-548d, miR-564, miR-572 and miR-652 ↓ miR-432	Lai et al., 2011
	115 SCZ vs. 40 CTRL	Serum	qRT-PCR	↑ let-7g, miR-181b, miR-219-2-3p, miR-1308 ↓ miR-195	Shi et al., 2012
	40 first-episode SCZ	Plasma	qRT-PCR	↓ miR-365 and miR-520c-3p after a 1-year treatment with risperidone	Liu et al., 2013
Bipolar disorder	21 BD vs. 21 CTRL	Plasma	qRT-PCR	In BD vs. ctrl: ↓ miR-134 ↑ miR-134 after a 4-weeks treatment (different combinations of antypsychotics and/or mood stabilizers)	Rong et al., 2011
Major depression	10 MD	Peripheral blood	TaqMan Array Human MicroRNA A+B Cards Set v. 3.0	30 miRNAs differentially expressed after a 12-weeks treatment with escitalopram: ↑ let-7d, let-7e, let-7f, let-7g, miR-22*, miR-26a, miR-26b, miR-29b-2*, miR-30d, miR-103, miR-106b*, miR-128, miR-130b*, miR-132, miR-140-3p, miR-183, miR-191, miR-335, miR-361-5p, miR-374b, miR-494, miR-500, miR-502-3p, miR-505*, miR-574-3p, miR-589, miR-629, miR-664 ↓ miR-770-5p, miR-34c-5p	Bocchio-Chiavetto et al., 2013
	9 MD vs. 9 CTRL	PBMCs	TaqMan Array Human MicroRNA A+B Cards Set v. 3.0	In MD vs. ctrl: ↑ miR-107, miR-133a, miR-148a, miR-425-3p, miR-494, miR-579, miR-589, miR-652, miR-941 ↓ miR-200c, miR-381, miR-517b, miR-636, miR-1243 After an 8-weeks treatment (different classes of antidepressant drugs): ↑ miR-20b-3p, miR-133a, miR-145, miR-409-3p, miR-410, miR-433, miR-485-3p ↓ miR-331-5p	Belzeaux et al., 2012
	40 MD vs. 40 CTRL	Serum	qRT-PCR	↑ miR-132 and miR-182	Li et al., 2013

*BD, bipolar disorder patients; CTRL, healthy controls; MD, major depression patients; PBMCs, peripheral blood mononuclear cells; SCZ, schizophrenia patients; WBCs, white blood cells*.

### Expression studies in neurologic disorders

#### Alzheimer's disease and other dementias

Because of its proximity to the brain parenchyma and the free exchange with the brain extracellular space, the biochemical composition of CSF provides information of the brain chemistry; this has determined the introduction of CSF biomarker analysis into routine clinical practice for AD (Blennow and Zetterberg, 2013). A first study conducted in CSF from AD patients identified 60 miRNAs as differentially expressed compared to healthy individuals, both upregulated and downregulated (Cogswell et al., 2008). Interestingly, these AD-specific miRNAs are linked to immunity-related pathways, in particular innate immunity and T cell activation and differentiation, which have been widely described to be altered in AD (Boutajangout and Wisniewski, 2013; Monsonego et al., 2013). In 2012, other CSF-derived miRNAs were described as differentially expressed in AD (Alexandrov et al., 2012), but contrasting results were reported by a recent study that showed an opposite alteration of some of the same miRNAs (Kiko et al., 2013). An interesting finding concerns the role played by let-7b, which was found to be increased in AD subjects. The intrathecal injection of let-7b into the CSF of mice resulted in neurodegeneration, an effect thought to be due to the activation of toll-like receptor (TLR) 7, since knock-out mice lacking TLR7 were resistant to neurodegeneration (Lehmann et al., 2012). Finally, a downregulation of miR-146 was detected in CSF from AD patients (Müller et al., 2014).

However, CSF is a not a readily accessible tissue and this may restrict the study sample sizes; to overcome these limitations, a number of researches was grounded on the analysis of peripheral blood and its derived products, which can be more easily obtained and potentially enable researchers to achieve larger samples. A first global investigation conducted on PBMCs led to the identification of 4 miRNAs upregulated in AD patients (Schipper et al., 2007). Still in PBMCs, a downregulation of miR-590-3p was described. Intriguingly, this miRNA is strongly predicted to target the heterogeneous nuclear ribonucleoprotein (hnRNP) A1, which is involved in the maturation of amyloid precursor protein (APP). The mRNA levels of hnRNPA1 were observed to be negatively correlated with miR-590-3p levels, supporting the hypothesis that this miRNA acts as a regulator of hnRNPA1, therefore influencing APP production (Villa et al., 2011). Another study on candidate miRNAs, previously found to be reduced in post-mortem brain cortices of AD patients (Geekiyanage and Chan, 2011), described a downregulation of 4 of these (among them, miR 29a/b) in the serum of individuals suffering from mild cognitive impairment (MCI) or AD (Geekiyanage et al., 2012). This consistency of results indicates that peripheral blood and its derivatives represent valid tissues to study miRNAs in CNS diseases, as they could reflect brain alterations. Interestingly, miR-29a/b had been previously shown to target BACE1/beta-secretase, which mediates the cleavage of APP producing β-amyloid peptide (Hébert et al., 2008). A serum alteration of 3 miRNAs was reported by an independent study on a wide sample of AD patients and controls (105 vs. 150 subjects) (Tan et al., 2014). Sheinerman et al. (2012) identified in plasma two sets of miRNA pairs differentiating early AD and MCI patients from healthy controls with good sensitivity and specificity. Finally, a very recent study employed a next-generation sequencing (NGS) technique to screen the entire miRNome in whole peripheral blood from AD patients (Leidinger et al., 2013). Through this comprehensive approach, 140 miRNAs were identified as differentially expressed in AD patients vs. control subjects. Moreover, a panel of 12 miRNAs (see Table 2) allowed to distinguish with high diagnostic accuracy (sensitivity and specificity >92%) between AD patients and healthy controls, and also between AD patients and patients affected by other neuropsychiatric disorders, including MCI, PD, as well as SCZ, MD, and BD.

**Table 2:** **microRNA expression studies in neurologic disorders**.

**Disease**	**Sample**	**Tissue**	**Method**	**Main finding**	**Reference**
Alzheimer's disease	10 AD vs. 10 CTRL	CSF	qRT-PCR	60 miRNAs differentially expressed; in the first positions: ↑ let-7f, miR-105, miR-125a, miR-135a, miR-138 ↓ miR-10a, miR-10b, miR-126, miR-126*, miR-127	Cogswell et al., 2008
	6 AD vs. 6 CTRL	CSF	Fluorescent miRNA array, LED-Northern dot blot	↑ miR-9, miR-125b, miR-146a, miR-155	Alexandrov et al., 2012
	13 AD vs. 11 CTRL	CSF	qRT-PCR	↑ let-7b	Lehmann et al., 2012
	20 AD vs. 22 CTRL	CSF	qRT-PCR	↓ miR-146	Müller et al., 2014
	10 AD vs. 10 CTRL	CSF, plasma	qRT-PCR	In CSF: ↓ miR-34a, miR-125b, miR-146a ↑ miR-29a, miR-29b In plasma: ↓ miR-34a, miR-146a	Kiko et al., 2013
	16 AD vs. 16 CTRL	PBMCs	MMChip, qRT-PCR	↑ miR-34a, miR-181b, miR-200a, let-7f	Schipper et al., 2007
	287 AD vs. 344 CTRL	PBMCs	qRT-PCR	↓ miR-590-3p	Villa et al., 2011
	7 AD, 7 MCI vs. 7 CTRL	Serum	qRT-PCR	↓ miR-137, miR-181c, miR-9, miR-29a/b	Geekiyanage et al., 2012
	105 AD vs. 150 CTRL	Serum	qRT-PCR	↓ miR-125b, miR-181c ↑ miR-9	Tan et al., 2014
	20 early AD, 20 MCI vs. 20 CTRL	Plasma	qRT-PCR	Two sets of miRNA pairs differentiating early AD and MCI from controls: 1) miR-128/miR-491-5p, miR-132/miR-491-5p and mir-874/miR-491-5p 2) miR-134/miR-370, miR-323-3p/miR-370 and miR-382/miR-370	Sheinerman et al., 2012
	106 AD vs. 22 CTRL	Peripheral blood	NGS (with Illumina HiSeq 2000), qRT-PCR	↑ brain-miR-112, brain-miR-161, let-7d-3p, miR-5010-3p, miR-26a-5p, miR-1285-5p, miR-151a-3p ↓ miR-103a-3p. miR-107, miR-532-5p, miR-26b-5p, let-7f-5p	Leidinger et al., 2013
Parkinson's disease	15 PD vs. 8 CTRL	Peripheral blood	qRT-PCR	In PD vs. ctrl: ↓ miR-1, miR-22*, miR-29 In treated with levodopa/carbidopa vs. untreated PD: ↑ miR-16-2*, miR-26a-2*	Margis et al., 2011
	19 PD vs. 13 CTRL	PBMCs	miRCURY LNA microRNA Array v. 10.0, qRT-PCR	↓ miR-116*, miR-32, miR-101 ↑ miR-15	Martins et al., 2011
	7 PD vs. 6 CTRL	WBCs	RNA-seq (with Applied Biosystems SOLiD sequencer)	In PD vs. ctrl: ↓ miR-320a/b/c, miR-769, miR-92b, miR-16 ↑ miR-199b, miR-1274b, miR-21, miR-150, miR-671, miR-1249, miR-20a, miR-18b*, miR-378c, miR-4293 In PD after DBS: ↓ miR-4293, miR-378c, miR-18b*, miR-20a, miR-1249, miR-424*, miR-210, miR-93 ↑ miR-4317, miR-143, miR-424	Soreq et al., 2013

*AD, Alzheimer's disease patients; CSF, cerebrospinal fluid; CTRL, healthy controls; MCI, mild cognitive impairment patients; PBMCs, peripheral blood mononuclear cells; PD, Parkinson's disease patients; WBCs, white blood cells*.

#### Parkinson's disease

A first study conducted on peripheral blood from PD patients revealed a decrease in the expression levels of 3 miRNAs; moreover, subjects treated with levodopa/carbidopa vs. untreated showed higher levels of other 2 miRNAs (Margis et al., 2011). A subsequent study analyzing miRNA expression profiles in PBMCs identified 4 differentially-expressed miRNAs. Interestingly, many of the predicted target genes revealed an overepresentation in pathways previously linked to PD, as well as in novel pathways (Martins et al., 2011). Finally, through RNA-seq other miRNAs were discovered to be differentially expressed in leukocytes from PD patients and, consistently, after deep brain stimulation (DBS) some of them were modulated in the opposite direction (Soreq et al., 2013).

Table 2 summarizes the above-reported expression studies in neurological disorders, with indication of samples, methodologies, and main results.

### Clues from expression studies: converging results and methodological issues

On the basis of the above-reported results on patients' peripheral matrices, a differential expression of a set of miRNAs emerges, supporting a role for miRNAs as key common players for different psychiatric and neurologic diseases. Moreover, many of these observations converge with results obtained in cerebral tissues from both humans and preclinical models.

**miR-134** was reported to be decreased in PBMCs and plasma respectively of SCZ and BD patients (Rong et al., 2011; Gardiner et al., 2012); its alteration, although with an opposite direction, was observed also in SCZ post-mortem brains (Santarelli et al., 2011), supporting a role of this miRNA in the illness pathogenesis. Moreover, miR-134 was increased within a set of miRNA associated to AD and MCI diagnosis in comparison to control subjects (Sheinerman et al., 2012). miR-134 is a brain actively regulated miRNA mainly localized in dendritic spines, with a major role in the regulation of synaptic proteins and neuronal plasticity, in terms of memory and cognitive functions, through a CREB-BDNF mediated mechanism (Gao et al., 2010a; Jimenez-Mateos et al., 2012; Bicker et al., 2013). Moreover, miR-134 is a fine-tuning regulator of embryonic neurodevelopment and neuronal differentiation both *in vitro* and *in vivo* (Gaughwin et al., 2011).

**miR-26a** and **miR-26b** were found altered in peripheral blood of MD patients during antidepressant treatment (Bocchio-Chiavetto et al., 2013), in AD patients (Leidinger et al., 2013) and PD patients (Margis et al., 2011), as well as in students experiencing pre-examination stress (Honda et al., 2013). Both miRNAs can regulate the expression of the neurotrophin BDNF, a main player of adult brain neurogenesis and synaptic plasticity maintenance (Caputo et al., 2011). A dysregulation of miR-26b was observed also in SCZ and AD post-mortem brains (Perkins et al., 2007; Absalon et al., 2013).

An increase in **miR-34a** peripheral blood cell content was observed in SCZ (Lai et al., 2011) and AD patients (Schipper et al., 2007), while antidepressant treatments were able to decrease **miR-34c**-5p in the blood of MD patients (Bocchio-Chiavetto et al., 2013). miR-34c levels were elevated in the hippocampus of AD patients and corresponding mouse models, suggesting that this miRNA could be a marker for the onset of cognitive disturbances (Zovoilis et al., 2011). In contrast, a downregulation of miR-34 was reported in CSF and plasma from AD patients (Kiko et al., 2013). Moreover, an alteration of miR-34a was evidenced also in the PFC of SCZ post-mortem brains (Kim et al., 2010). Basic studies indicated that Drosophila miR-34 has a role in age-associated events, aging, and neurodegeneration (Liu et al., 2012a). Experiments with antagomiRs revealed that targeting miR-34a might increase neuronal survival and reduce death and apoptosis in a rat model of temporal lobe epilepsy (Hu et al., 2012). Moreover, *in vitro* experiments showed that ectopic expression of miR-34a downregulates the endogenous activity-regulated, cytoskeleton-associated protein Arc, a crucial factor for experience-dependent synaptic plasticity and long-term memory in mammals (Wibrand et al., 2012). Finally, studies in mice models indicated a role for miR-34a in the central stress response and suggested this miRNA as a potential target for the treatment of stress-related disorders (Haramati et al., 2011).

**miR-107** was found increased in PBMCs from MD subjects (Belzeaux et al., 2012), but decreased in PBMCs of SCZ (Gardiner et al., 2012) and in blood of AD patients (Leidinger et al., 2013). Reduced levels of miR-107 were found also in AD post-mortem brains (Nelson and Wang, 2010) and a recent study in SCZ post-mortem brains correlated the expression levels of miR-107 with a loss in the expression of cortical muscarinic receptors (CHRM1), observed in the 25% of patient tissues (Scarr et al., 2013). Moreover, altered miR-107 were associated with cytoskeletal pathology in a transgenic mouse model of AD and with granulin/progranulin expression regulation *in vivo* and *in vitro*, with implications for brain disorders (Wang et al., 2008a, 2010b).

miRNAs of the let-7 family (**let-7b**, **let-7d-3p**, **let-7f**, **let-7g**) were found dysregulated in different peripheral tissues of SCZ patients (Shi et al., 2012), AD patients (Schipper et al., 2007; Cogswell et al., 2008; Lehmann et al., 2012; Leidinger et al., 2013) and modulated by antidepressant treatment (Bocchio-Chiavetto et al., 2013), supporting their involvement in mental disorder etiology and treatment. In this regard, studies in animal models indicated a neurodegenerative effect of let-7b (Lehmann et al., 2012) and a negative regulation of the cortical muscarinic acetylcholine receptor levels (M1) (Creson et al., 2011).

Other studies indicated an increase of **miR-181b** in serum of SCZ patients (Shi et al., 2012) and in PBMCs of AD patients (Schipper et al., 2007). In parallel, an upregulated expression of this miRNA was reported in the temporal cortex of SCZ post-mortem brains, with a concomitant downregulation of its main neural target genes, the calcium sensor gene visinin-like 1 (VSNL1) and the ionotropic AMPA glutamate receptor subunit (GRIA2), suggesting possible effects on gene expression in patients (Beveridge et al., 2008). A role of miR-181b in mental pathologies could be also linked to its involvement in neuroprotection (Peng et al., 2013) and in NMDA receptor-dependent plasticity response in mature neurons (van Spronsen et al., 2013).

Elevated levels of **miR-9** were reported in the CSF of AD patients (Alexandrov et al., 2012), whereas lower levels were observed in serum of patients with the same pathology (Geekiyanage et al., 2012). miR-9 is widely expressed in the mammalian brain and can play a role in different neuronal functions, ranging from early neurogenesis and differentiation to dendritic morphogenesis and synaptic plasticity in the adult brain (Gao, 2010b), as well as in neurotoxic mechanisms, since the expression of miR-9 is downregulated by β-amyloid in hippocampal cell cultures (Schonrock et al., 2010).

Finally, alterations in peripheral levels of **miR-132** were recently associated to MD and AD/MCI diagnosis (Sheinerman et al., 2012; Li et al., 2013), as well as to the effects of antidepressant therapy (Bocchio-Chiavetto et al., 2013). These data are consistent with the observations obtained in AD and FTLD post-mortem brains (Hébert et al., 2013; Lau et al., 2013), confirming in patients' tissues the substantial role played by miR-132 in basic mechanisms of synaptic plasticity. In particular, miR-132 is one of the main mediators of the beneficial effects of the neurotrophin BDNF on CNS neurons (Numakawa et al., 2011) and it is implicated in brain response to stress stimuli (Shaltiel et al., 2013), as well as in the regulation of cognitive function as learning and memory formation (Hansen et al., 2013).

Generally speaking, miRNAs in body fluids were measured both as single candidates using qRT-PCR methods and by the employment of “whole-genome” approaches, through different miRNA profiling techniques. In the reviewed studies, the most used technologies were microarrays for the simultaneous analysis of, at most, about 900 miRNAs (Schipper et al., 2007; Alexandrov et al., 2012; Gardiner et al., 2012) and qRT-PCR arrays which can detect about 750 miRNAs (Belzeaux et al., 2012; Bocchio-Chiavetto et al., 2013). Microarray technologies permit a lower cost miRNA profiling, compared to qRT-PCR arrays, but they require the subsequent validation of the most significant results through qRT-PCR; this can be money- and time-consuming, particularly in large study samples. Two studies (Leidinger et al., 2013; Soreq et al., 2013) analyzed miRNA profiles with small RNA NGS, which virtually allows the detection of all the miRNAs and other small RNAs expressed in a given sample. NGS techniques were employed also in basic examinations to characterize miRNA whole expression in different biofluids, highlighting the presence of more than 500 and 400 miRNAs, respectively in serum and CSF of healthy subjects (Burgos et al., 2013).

It is still an open question whether and how miRNA levels in the periphery (such as in CSF, serum, plasma, blood, lymphoblasts etc.) may reflect brain modifications, and this issue generally concerns all the potential peripheral biomarkers in psychiatric and neurologic disorders. A first evidence has suggested a possible correlation between central and peripheral levels, since miRNAs could pass through biological membranes in free-form or into microvesicles. In this regard, tumor-specific microvesicles containing miRNAs at altered levels were detected in the serum of patients affected by glioblastomas (Skog et al., 2008) and brain-specific miRNAs quantified in plasma were proposed as biomarkers for brain injury in animal models (Laterza et al., 2009). Finally the levels of miR-210, described as significantly decreased in the blood of stroke patients, showed a correlation between brain and blood in ischemic mice (Zeng et al., 2011).

A major reason for the lack of conclusive data might be attributed to the low number of studies which have explored brain-periphery correlation, also due to the difficulties in getting concomitant brain and peripheral samples of the same human subjects. This problem could be overcome by the employment of animal models; however, these studies are limited, since the number of annotated miRNAs is widely different between species (miRBase 20th release, June 2013: 2578 mature miRNAs in humans, 728 in rats and 1908 in mice).

## Genetic variants in microRNA-related genes in psychiatric and neurologic disorders

### Single nucleotide polymorphisms in microRNA mature sequences or precursors

The most considerable findings about an involvement of SNPs located in miRNA mature sequences or precursors in neuropsychiatric disorders come from studies on SCZ. Hansen et al. (2007) identified an association between a SNP located in the brain-expressed mir-206 (rs17578796) and the disease. Few years later, an analysis of SNPs in miRNAs mapping on the X chromosome, conducted on male SCZ subjects, led to the identification of 8 ultra-rare variants in 8 distinct miRNA genes (3 precursor and 5 mature miRNA sequences) in 4% of the analyzed patients (Feng et al., 2009). In a Chinese population, a SNP located in pre-mir-30e (ss178077483) was later detected to be associated to SCZ (Xu et al., 2010a), and the same research group described an association of this variant also with MD (Xu et al., 2010b). In a GWAS of substantial size on SCZ, the strongest finding was with rs1625579, a SNP located within an intron of the non-protein coding gene AK094607, which contains the primary transcript for miR-137, a known regulator of neuronal development (Ripke et al., 2011). However, the polymorphism resides more than 8 kilobases away from the pri-miR-137. In subsequent studies the same variant was associated with specific SCZ/psychosis endophenotypes, characterized by severe cognitive deficits and negative symptoms, rather then with the disease itself (Cummings et al., 2013; Green et al., 2013). Another recent study suggested a possible functional explanation for this SNP, showing an association between the risk genotype and reduced expression levels of miR-137 in the dorsolateral PFC of healthy subjects; interestingly, this corresponded to increased levels of the miR-137 target gene TCF4, a SCZ candidate gene (Guella et al., 2013).

Small evidence is available for MD; in addition to the above-mentioned study on pre-mir-30e (ss178077483) (Xu et al., 2010b), so far only another investigation has been conducted, revealing an association between a SNP in pre-mir-182 and late insomnia in MD patients, thus suggesting that this variant could be involved in the alteration of circadian rhythms described in depressed patients (Saus et al., 2010).

Finally, two SNPs, respectively located in mir-22 (rs6502892) and mir-339 (rs11763020), were associated to panic disorder. Interestingly, functional studies showed that mir-22 is a regulator of candidate genes for panic disorder (BDNF, HTR2C, MAOA and RGS2), suggesting a possible contribution of its genetic variants in the development of this disease (Muiños-Gimeno et al., 2011).

### Single nucleotide polymorphisms in microRNA target genes

Genetic variants in miRNA target genes, in particular in their 3'-UTR, are equally important as SNPs in miRNA mature sequences or precursors, since they can alter the complementarity between mRNA and miRNA, therefore influencing their binding.

Concerning psychiatric diseases, a SNP (rs3822674) in the complexin 2 gene (CPLX2), associated with altered cognition in SCZ subjects, was described to affect miR-498 binding and gene expression (Begemann et al., 2010). Interestingly, an allelic variant of rs11122396 in the 3'-UTR of disrupted-in-schizophrenia-1 (DISC-1) gene, which had been associated to schizophrenia through a rare haplotype (Hennah et al., 2003), has recently been brought to the forefront thanks to a functional study showing that this variant disrupts miR-135b-5p binding, leading to elevated DISC-1 levels (Rossi et al., 2013). Finally, 3 other SNPs (rs17110432, rs11178988 and rs11178989) in the 3'-UTR of TBC1D15 gene were reported as associated to SCZ and predicted to affect miRNA binding (Liu et al., 2012b).

Another significant association was detected between allelic variants of rs1653625 in the purinergic receptor P2X gene (P2RX7) and MD; this SNP resides in a putative miRNA target site (Rahman et al., 2010). In patients affected by obsessive-compulsive disorder (OCD), a SNP (rs28521337) located in a functional target site for miR-485-3p, in the truncated isoform of neurotrophin-3 receptor gene (NTRK3), was associated with hoarding, a particular endophenotype of the disease (Muiños-Gimeno et al., 2009).

Evidence in this field is available also for neurodegenerative diseases: a study conducted on FTLD with TDP-43 inclusions (FTLD-TDP) by Rademakers et al. (2008) unveiled a functional effect for the high-risk allele of rs5848 in progranulin gene (GRN), which promotes a more efficient binding of miR-659, resulting in augmented translational inhibition of GRN. Similarly, rs1050283 in the oxidized LDL receptor 1 gene (OLR1), which acts as a risk factor for sporadic AD, was hypothesized to influence miR369-3p binding (Serpente et al., 2011). Moreover, other previously known AD-associated genetic variants in the 3'-UTR of APP were empirically shown to influence miRNA binding, both by inhibiting (T117C, effect on miR-147) or increasing it (A454G, effect on miR-20a), and therefore inversely affecting APP levels (Delay et al., 2011).

An association was also detected between PD and rs12720208 in the 3'-UTR of fibroblast growth factor 20 (FGF20) gene. The risk allele was described to disrupt a binding site for miR-433, increasing FGF20 levels *in vitro* and *in vivo*, and the increase was correlated with α-synuclein overexpression, which had previously been implicated in PD pathophysiology (Wang et al., 2008b), though a later study failed to confirm the association of this SNP with PD (de Mena et al., 2010).

### Single nucleotide polymorphisms in microRNA processing genes

Genetic variants affecting genes implicated in miRNA biogenesis and processing are extremely relevant, since they can exert pleiotropic effects on a multitude of miRNAs.

So far, only two studies have inquired into this important topic; the first one investigated the possible association between MD and 3 SNPs located in 3 miRNA processing genes (DGCR8, DICER, and GEMIN4). Variants of rs3757 in DGCR8 and rs636832 in AGO1 were associated with an increased risk for this disease (He et al., 2012). The second study evaluated 6 SNPs in 5 miRNA processing genes (DGCR8, DICER, GEMIN4, DROSHA, and AGO1) in SCZ subjects. The same above-mentioned variant in DGCR8 was found to be associated also with SCZ, together with rs3742330 in DICER (Zhou et al., 2013). Interestingly, an elevated expression of DGCR8 was observed in PFC of SCZ patients (Santarelli et al., 2011); rs3757 is located in the 3'-UTR of this gene, possibly affecting the regulation of its expression by miRNAs and resulting in an overall increase in miRNA production.

Table 3 summarizes all the above-reported genetic studies in psychiatric and neurologic disorders, with indication of samples and main results.

**Table 3:** **Genetic studies on microRNA-related genes in psychiatric and neurologic disorders**.

	**Sample**	**Main finding**	**Reference**
SNPs in miRNAs	840 SCZ vs. 1476 CTRL (Scandinavian)	rs17578796 (in mir-206) associated with SCZ	Hansen et al., 2007
	193 SCZ vs. 191 CTRL (Caucasian, all males)	Eight ultra-rare variants on X chromosome identified in 4% of SCZ patients: 32 A>G in pre-mir-18b, 8 C>T in pre-mir-505, 13 C>G in pre-mir-502, 11 G>A in let-7f-2, 7 C>T in mir-188-3p, 8 G>A in mir-325-3p, 15 C>T in mir-660, 13 C>T in mir-509-3p	Feng et al., 2009
	456 SCZ vs. 453 CTRL (Chinese)	ss178077483 (in pre-mir-30e) associated with SCZ	Xu et al., 2010a
	Discovery stage: 9394 SCZ vs. 12462 CTRL; replication stage: 8442 SCZ vs. 21397 CTRL (European-ancestry)	rs1625579 (in a putative primary transcript for mir-137) associated with SCZ	Ripke et al., 2011
	617 SCZ vs. 764 CTRL (Australian)	rs1625579 (in a putative primary transcript for mir-137) associated with a specific SCZ phenotype	Green et al., 2013
	821 SCZ/BD/SCHIZOAFFECTIVE vs. 171 CTRL (Irish)	rs1625579 (in a putative primary transcript for mir-137) associated with a specific psychosis phenotype	Cummings et al., 2013
	1088 MD vs. 1102 CTRL (Chinese)	ss178077483 (in pre-mir-30e) associated with MD	Xu et al., 2010b
	359 MD vs. 341 CTRL (Spanish)	rs76481776 (in pre-mir-182) associated with late insomnia MD	Saus et al., 2010
	200 panic disorder vs. 340 CTRL (Spanish)	rs6502892 (in mir-22) and rs11763020 (in mir-339) associated with panic disorder	Muiños-Gimeno et al., 2011
SNPs in miRNA target genes	1071 SCZ (Caucasian)	rs3822674 (in CPLX2 gene) associated with altered cognition in SCZ subjects, affecting miR-498 binding and gene expression	Begemann et al., 2010
	607 SCZ vs. 1128 CTRL (Finnish)	rs11122396 in DISC-1 gene associated to SCZ through a rare haplotype	Hennah et al., 2003
	746 SCZ vs. 1599 CTRL (Chinese)	rs17110432, rs11178988 and rs11178989) in TBC1D15 gene associated to SCZ, possibly affecting miRNA binding	Liu et al., 2012b
	171 MD+BD vs. 178 CTRL (Hungarian)	rs1653625 (in a putative miRNA-target site in P2RX7 gene) associated with MD	Rahman et al., 2010
	153 OCD (Spanish)	rs28521337 (in a target site for miR-485-3p in the truncated isoform of NTRK3 gene) associated with hoarding phenotype of OCD	Muiños-Gimeno et al., 2009
	59 FTLD vs. 433 CTRL (ethnicity not specified)	rs5848 (in GRN gene) associated with FTLD, enhancing miR-659 binding and translational inhibition of GRN	Rademakers et al., 2008
	453 AD vs. 393 CTRL (Italian)	rs1050283 (in OLR1 gene) associated with AD, possibly influencing miR-369-3p binding	Serpente et al., 2011
	1089 PD vs. 1165 CTRL (ethnicity not specified)	rs12720208 (in FGF20 gene) associated with PD, disrupting miR-433 binding and increasing FGF20 levels	Wang et al., 2008b
SNPs in miRNA processing genes	252 SCZ vs. 256 CTRL (Chinese)	rs3757 (in DGCR8 gene) and rs3742330 (in DICER gene) associated with SCZ	Zhou et al., 2013
	314 MD vs. 252 CTRL (Chinese)	rs3757 (in DGCR8 gene) and rs636832 (in AGO1 gene) associated with MD	He et al., 2012

*BD, bipolar disorder patients; CTRL, healthy controls; FTLD, frontotemporal lobar degeneration; MD, major depression patients; OCD, obsessive-compulsive disorder patients; SCZ, schizophrenia patients*.

## Conclusions

The research on miRNA involvement in psychiatric and neurologic disorders has grown up in the last years, supporting a role of miRNAs in several neuropsychiatric conditions and suggesting a possible usefulness of these small non-coding RNAs as disease-related biomarkers.

However, the continuously growing number of annotated miRNAs, as described in Figure 1, implies a major complexity in the global interpretation of the current available results, since many newly discovered miRNAs have not been sufficiently studied yet. New investigations will be welcome to clarify the role played by the already known miRNAs and to identify new critical ones. Moreover, some technical issues have to be resolved. A reliable and reproducible quantification of miRNAs is essential to compare results arising from different studies and, given that many experimental variables can affect miRNA measurement, all the related technical procedures should be carefully optimized and standardized. Till then, the transferability of these studies to the clinical practice will be inevitably limited. Finally, there is still a lack of data about the origin of miRNAs in blood and also whether they accurately reflect miRNA activity in the brain; further studies to elucidate these aspects are needed (Kolshus et al., 2013).

Concerning genetic studies on miRNA-related genes, we are still in an embryonic stage, but the recent annotation of new miRNA SNPs paves the way to a growing research in this field. The study of miRNA CNVs is an even more unexplored area; new investigations on these topics are strongly advisable to widen the knowledge on the genetic bases of psychiatric and neurologic diseases. Great help for these investigations is likely to come from the new NGS technologies, allowing a faster and cheaper scan of the whole genome than ever before.

In general, the scientific community has great expectations for the use of miRNA measures and genetic data as non-invasive biomarkers for the diagnosis, prognosis, and therapeutic appraisal of many illnesses. The fact that differential expression levels of peripheral miRNAs have been associated with several disease processes and to similar modifications in brain tissues suggests the potential for using them as a new generation of biomarkers in neuropsychiatric conditions and opens new avenues for the treatment of these disorders.

### Conflict of interest statement

The authors declare that the research was conducted in the absence of any commercial or financial relationships that could be construed as a potential conflict of interest.
